# Characteristics of *N*-Acylhomoserine Lactones Produced by *Hafnia alvei* H4 Isolated from Spoiled Instant Sea Cucumber

**DOI:** 10.3390/s17040772

**Published:** 2017-04-05

**Authors:** Hong-Man Hou, Yao-Lei Zhu, Jia-Ying Wang, Feng Jiang, Wen-Yan Qu, Gong-Liang Zhang, Hong-Shun Hao

**Affiliations:** 1 School of Food Science and Technology, Dalian Polytechnic University, No. 1, Qinggongyuan, Ganjingzi District, Dalian 116034, China; yaoleizhu@126.com (Y.-L.Z.); wangjiayingle@163.com (J.-Y.W.); jiangfeng656991@163.com (F.J.); wenyanq@163.com (W.-Y.Q.); zhanggl1978@hotmail.com (G.-L.Z.); 2 Liaoning Key Lab for Aquatic Processing Quality and Safety, No. 1, Qinggongyuan, Ganjingzi District, Dalian 116034, China; beike1952@163.com

**Keywords:** *Hafnia alvei*, quorum sensing, AHLs, instant sea cucumber, biofilm formation

## Abstract

This study aimed to identify *N*-acylhomoserine lactone (AHL) produced by *Hafnia alvei* H4, which was isolated from spoiled instant sea cucumber, and to investigate the effect of AHLs on biofilm formation. Two biosensor strains, *Chromobacterium violaceum* CV026 and *Agrobacterium tumefaciens* KYC55, were used to detect the quorum sensing (QS) activity of *H. alvei* H4 and to confirm the existence of AHL-mediated QS system. Thin layer chromatography (TLC) and high resolution triple quadrupole liquid chromatography/mass spectrometry (LC/MS) analysis of the AHLs extracted from the culture supernatant of *H. alvei* H4 revealed the existence of at least three AHLs: *N*-hexanoyl-l-homoserine lactone (C6-HSL), *N*-(3-oxo-octanoyl)-l-homoserine lactone (3-oxo-C8-HSL), and *N*-butyryl-l-homoserine lactone (C4-HSL). This is the first report of the production of C4-HSL by *H. alvei*. In order to determine the relationship between the production of AHL by *H. alvei* H4 and bacterial growth, the β-galactosidase assay was employed to monitor AHL activity during a 48-h growth phase. AHLs production reached a maximum level of 134.6 Miller unites at late log phase (after 18 h) and then decreased to a stable level of about 100 Miller unites. AHL production and bacterial growth displayed a similar trend, suggesting that growth of *H. alvei* H4 might be regulated by QS. The effect of AHLs on biofilm formation of *H. alvei* H4 was investigated by adding exogenous AHLs (C4-HSL, C6-HSL and 3-oxo-C8-HSL) to *H. alvei* H4 culture. Biofilm formation was significantly promoted (*p* < 0.05) by 5 and 10 µM C6-HSL, inhibited (*p* < 0.05) by C4-HSL (5 and 10 µM) and 5 µM 3-oxo-C8-HSL, suggesting that QS may have a regulatory role in the biofilm formation of *H. alvei* H4.

## 1. Introduction

Food spoilage is a major socio-economic problem that occurs mainly as a result of the biochemical activity of a microbial community that renders a product undesirable or unacceptable for consumption [1]. Moreover, food spoilage is mostly caused by bacteria, and quorum sensing (QS) is thought to play a role in bacterial-linked food spoilage and food safety [2,3,4]. Increased interest in the connection between QS and food spoilage has led to more in-depth study of the signal molecules in relation to microbes that cause food spoilage. Several classes of signaling molecules of microbial origin, especially *N*-acyl homoserine lactones (AHLs), have now been identified and detected in different kinds of food, such as milk, meat, and vegetables [5,6].

AHLs are a kind of fatty acid derivatives that are generally recognized as autoinducer-1 (AI-1). These compounds are secreted by Gram-negative bacteria and used as the signals for intraspecies communication [7]. The structure of AHLs consist of a homoserine lactone ring, which is *N*-acylated, and a fatty acyl group linked to the position of C-1, with the acyl chains typically ranging from four to 18 carbons [8]. Bacteria are known to produce several kinds of AHLs, and one type of AHL can also be synthesized by various genera [1]. Several environmental conditions, including temperature, pH, and NaCl, may influence the concentrations and types of AHLs being produced [9]. The amount of AHLs detected in rotten foods seems to correlate with the expression of certain proteases and the growth of spoilage bacteria [10].

In recent years, instant sea cucumber, which can be eaten without further preparation, is becoming more and more popular among sea cucumber consumers. These sea cucumbers can better maintain their nutritional qualities and have more beneficial amino acid composition compared to traditional dried sea cucumber [11]. Instant sea cucumbers are generally stored at 0–5 °C for three months, making them susceptible to spoilage caused mainly by bacteria. Microbial transmission occurring during food processing is a primary reason why food can become contaminated by microbes, and biofilm formation can benefit the process of this transmission [12,13]. Biofilms are aggregates of microorganisms within a matrix composed of extracellular biopolymers that adhere to a solid surface such as food, or food-processing equipment, resulting in reduced effectiveness of disinfectants. Decontamination of biofilms on food-processing equipment is particularly difficult as biofilms frequently slough off, releasing cells into contacted food products [14] and induce food spoilage. Therefore, biofilms play an important part in the spoilage of instant sea cucumber. Since QS molecules, it is important to know the effect they have on biofilm formation. Previous studies have verified the effect of AHLs on biofilm formed by *Pseudoalteromonas ulvae* TC14 and *Shewanella baltica* has been verified [15,16]. According to our best knowledge, there are few reports on QS and biofilm formation caused by *H. alvei* isolated from instant seafood.

*Hafnia alvei* is an opportunistic pathogen belonging to the *Enterbacteriaceae* family [17]. It is a Gram-negative bacterium and a common food contaminant capable of producing AHLs. In addition, *H. alvei* is considered as the most commonly isolated from contaminant vacuum-packed chilled meat samples [18]. Production of *N*-(3-oxohexanoyl) homoserine lactone (3-oxo-C6-HSL) by *H. alvei* has been reported by Viana et al. [19]. Furthermore, food spoilage and biofilm formation have been linked to the QS activity of *H. alvei*. In this study, three strains of bacteria (H2, H4, and H7) were isolated from spoiled instant sea cucumber and their ability to produce AHLs was assessed. H4 exhibited stronger AHL activity than the other two strains. It was identified as an *H. alvei* strain. Considering the link of QS to food spoilage and biofilm formation, the AHLs produced by *H. alvei* H4 were identified and their effect on biofilm formation was also investigated.

## 2. Experimental Section

### 2.1. Sample Collection and Bacterial Strains Isolation

The bacterial strain used in this study was isolated from spoiled instant sea cucumber. Each sample was cut into 25-g pieces and minced with sterile knife, then mixed with 225 mL of normal saline under sterile condition. The sample mixture was homogenized for 60 s and 100 μL samples were spread onto LB (10 g Tryptone, 5 g Yeast extract power, 10 g NaCl, dissolved in 1 L deionized water) agar plates as previously described [20]. The plates were incubated at 28 °C for 24 to 48 h. Several morphologically distinct colonies that appeared on the plates were randomly chosen and subcultured at 28 °C for 24 to 48 h to obtain a pure culture, which was then maintained on LB agar plate at 4 °C. 

### 2.2. Screening for Bacterial Isolates for AHLs Production

Two AHLs bacterial biosensors, *Chromobacterium violaceum* CV026 and *Agrobacterium tumefaciens* KYC55 which respond to short-chain and long-chain AHLs, respectively, were used in the preliminary screening of AHL produced by the bacteria isolated from spoiled instant sea cucumber. Agar plate diffusion assay was used in the AHL screening process that multiple AHLs-producting bacteria were screened in one culture dish by Anbazhagan et al. [21], to avoid the false positives due to possible contamination between these isolated strains in one dish, some modifications were carried out. Briefly, the bacterial strain to be tested for the production of AHLs as well as *C. violaceum* CV026 were streaked parallel to each other on a LB agar plate. Detection of AHLs production was also carried using *A. tumefaciens* KYC55, and the assay was basically performed in the same way*,* except that the agar was supplemented with 50 μg/mL X-gal (Sangon Biotech, Shanghai, China). In both cases, the plates were incubated at 28 °C for overnight. Production of exogenous AHLs was indicated by the formation of the purple pigment violacein or blue coloration by β-galactosidase activity.

### 2.3. Identification and Phylogenetic Analysis of AHL-Producing Bacteria

Genomic DNA of H2, H4, and H7 strains was extracted with TIANamp bacteria DNA kit (Tiangen Biotech, Beijing, China) and then used as template to amplify the 16S rDNA gene. The primers used were 27F (5′-AGAGTTTGATCMTGGCTCAG-3′) and 1492R (5′-GGTTACCTTGTTACGACTT-3′). The PCR sample contained 1 μL of each primer (10 μM), 1 μL DNA template solution, 10 μL Premix Ex Taq DNA polymerase buffer (Takara, Tokyo, Japan), and 7 μL ultra-pure water. PCR condition consisted of 95 °C 5 min; 30 cycles of 95 °C 40 s, 55 °C 1 min, and 72 °C 2 min, and a final elongation step at 72 °C 10 min. PCR products were purified and sequenced by BGI (Shenzhen, China). 16S rDNA sequences were used to identify bacterial genera by comparing the sequences with those in the GenBank database (http://blast.ncbi.nlm.nih.gov). To construct a phylogenetic tree based on its 16S rDNA sequence, CLUSTAL W [22] was used to align the nucleotide sequences of the AHL-producing bacterial isolates (http://www.genome.jp/tools/clustalw/). Molecular Evolutionary Genetic Analysis version 3.1 (MEGA3.1) was used to construct the phylogenetic tree, and the aligned complete 16S rDNA sequences were subjected to phylogenetic analysis using MEGA 3.1. To ensure the robustness and reliability of trees constructed, Neighbor-Joining algorithm method and bootstrap analysis for 500 resamplings were conducted to generate the tree [23].

### 2.4. Extraction of AHLs

In order to identify the AHLs produced by the isolated bacterial strain with strong QS activity from spoiled instant sea cucumber, AHLs were first extracted from the corresponding bacterial culture. Aliquot (100 mL) of an overnight culture was centrifuged at 8000× *g* for 15 min at 4 °C, and the supernatant was added to an equal volume of ethyl acetate containing 0.1% acetic acid (v/v) followed by thorough mixing. The mixture was then incubated at 25 °C for 2 h with shaking at 180 rpm. After that, the ethyl acetate layer was removed and freeze-dried under vacuum, while the residue was dissolved in 1 mL ultra-pure water, and then dried in a vacuum drier. The sample was dissolved in 100 μL 25/75 (v/v) acetonitrile/water and subjected to TLC and LC-MS analyses as described below.

### 2.5. Identification of AHLs by TLC and HPLC/MS

Reverse phrase C18 TLC plate (Merck, Darmstadt, Germany) was cut into a 4 × 10 cm strip, AHL extracted and standard AHLs (C4-HSL (100 μg/mL), C6-HSL (50 μg/mL) and 3-oxo-C8-HSL (100 μg/mL) obtained from Sigma–Aldrich (St. Louis, MI, USA) were spotted onto the strip about 1 cm from the bottom of the strip, and with a spacing of 1 cm between spots. Methanol/water (50%, v/v) was used as an eluting solvent to separate the AHLs. After development, the strip was air dried at 25 °C, and the dried strip was then put into a culture dish and overlaid with LB agar containing *C. violaceum* CV026. After setting, the plate was incubated at 28 °C for overnight. The presence of AHL would result in the appearance of purple spots in the agar, indicative of the production of a purple pigment by C. *violaceum* CV026 induced by AHL [24].

For mass spectrometry analysis, individual AHLs were first purified from the extract by solid phase extraction (SPE) as optimized by Li et al. [25] using Varian Bond Elut C18 (1 g, 6 mL) (Agilent Technolgies, Palo Alto, CA, USA). Identification of AHLs extracted from the bacterial culture were performed as described by Okutsu et al. [26], but with some modification, using a triple quadrupole/linear ion trap instrument (LIT) (QTRAP4000; AB Sciex, Foster, CA, USA) with an electrospray ionization (ESI) source equipped with a RRHD SB-C18 column (3.0 mm × 100 mm, 1.8 μm particle size) kept at 30 °C. The sample (6 μL) was loaded onto the column and eluted with acetonitrile and water, both of which contained 0.1% (v/v) acetic acid. The flow rate was set at 0.3 mL/min for 30 min, and the acetonitrile gradient was set from 30% (v/v) at 0 min to 70% (v/v) at 30 min. The eluent was monitored by absorbance at 210 nm. LIT was used to record the MS/MS spectra with product ion scan mode. Ion source was kept at 450 °C, curtain gas was at 10, collisional activated dissociation (CAD) gas at medium, ion source gas 1 at 30 psi, and ion source gas 2 at 30 psi. Ionspray voltage was set at 5500 V in positive ion mode. Declustering Potential was set at 100 V, Entrance Potential 10 V, Collision Potential 15 V, and Collision Potential cell exit potentials was maintained at 10 V. Positive ion mode was performed to scan precursor ion with Q1 set to scan a mass range from *m*/*z* 100 to 250 Da. In addition, Q3 was set at *m*/*z* 102 Da to monitor the lactone ring of the product ion. Standard AHLs were used as references: C6-HSL, 3-oxo-C8-HSL and C4-HSL (Sigma–Aldrich, St. Louis, MI, USA) and analyzed by HPLC/MS with the same settings described above.

### 2.6. AHLs Quantification by β-Galactosidase Assay

The quantity of AHLs produced by an AHL-positive strain was measured by determining the level of β-galactosidase activity with o-nitrophenyl-D-galactopyranoside (ONPG, Sigma–Aldrich, St. Louis, MI, USA) as a substrate as previously described [27]. Briefly, 100 µL sterile-filtered culture supernatant of an overnight culture of *H. alvei* H4 was added to 1 mL of diluted KYC55 culture (10^6^–10^7^ CFU/mL) and the mixture was inoculated at 30 °C until the OD_600_ of the culture reached 0.2–1.0. The cells were harvested from 200 μL culture and resuspended in 1 mL of Z buffer (60 mM Na_2_HPO4, 40 mM NaH_2_PO_4_, 10 mM KCl, 1 mM MgSO_4_, pH 7.0) followed by the addition of one drop of 0.1% SDS and three drops of chloroform to lyse the bacterial cells. The tubes were then placed in a 30 °C water bath and allowed to equilibrate for about 5 min. Then, 0.2 mL ONPG (final concentration of 4 mg/mL in 0.1 M phosphate buffer pH 7.0) was added to each tube followed by vortexing. The reaction was stopped by adding 0.6 mL of 1 M Na_2_CO_3_ after the appearance of a yellow color. The mixture was centrifuged at 12,000× *g* for 15 min and the absorbance of the supernatant was measured at 420 nm using a UV spectrophotometer. The activity of β-galactosidase was calculated as Equation (1) below. AHL production was monitored over a 48-h period, with measurement taken at every 6 h. For the negative control, 100 μL LB medium was used instead of sterile bacterial culture supernatant.

(1) $$\text{Miller unites}=\frac{A_{420}\times 1000}{T\times V\times A_{600}}$$

### 2.7. Biofilm Formation Assay

The quantification of biofilm formation was performed using a regular 96-well microtiter plates as described previously [28], but with minor modifications. Briefly, an overnight culture of *H. alvei* H4 was inoculated (1:100 dilution) in LB medium and 200 μL of the culture was added to a 96-well microtiter plate (Corning, Corning, NY, USA) followed by the addition of exogenous synthetic AHL (C4-HSL, C6-HSL or 3-oxo-C8-HSL; Sigma–Aldrich, St. Louis, MI, USA) to a final concentration of 5, 10, 20, or 40 μM. For control, deionized water was used instead of AHL. The plate was incubated at 30 °C for 48 h. After incubation, the culture suspension was discarded and the plate was washed three times with PBS (pH 7.4, 0.01 M) using 200 μL per well. The cells were then fixed with 200 μL methanol for 15 min, dried at 60 °C and stained with 200 μL of 0.1% crystal violet for 15 min. The plate was again washed three times with deionized water to remove the excess dye and then dried at 60 °C. Biofilm formed on the plate was solubilized by addition of 200 µL of 33% acetic acid (per well) followed by 20 min incubation at room temperature. The absorbance of the plate was then measured at 590 nm with a spectrophotometer (Molecular Devices, San Francisco, CA, USA).

### 2.8. Statistical Analysis

Each sample was conducted by three replicate trials, and all experiments were repeated three times. Results were presented as mean ± standard deviation (SD) and analyzed by T-test using SPSS 16.0 software and the graphics were made by origin pro8.6 version.

## 3. Result

### 3.1. Isolation and Identification of AHL-Producing Bacteria

**Figure 1:**
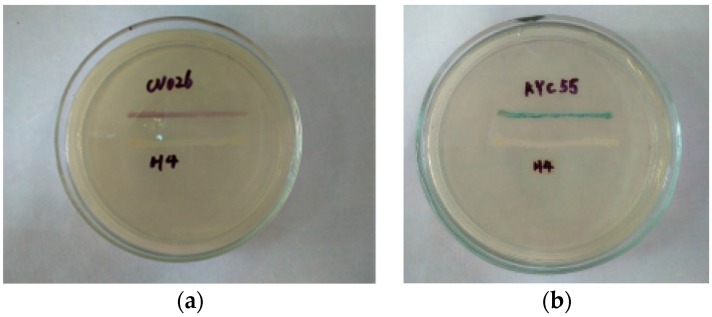
Screening of *N*-acylhomoserine lactone (AHL)–producing bacteria by (**a**) *C. violaceum* CV026 and (**b**) *A. tumefaciens* KYC55 strain. H4: bacterium isolated from spoiled sea cucumber; CV026 and KYC55: biosensor strains used to detect AHL-producing strains.

Three dominant food spoilage bacteria (H2, H4, and H7) were isolated from spoiled instant sea cucumber and their ability to produce AHLs was determined. AHL production was only detected for strain H4 when the assay was based on color changes produced by the reporter strains: purple in the case of *C. violaceum* 026 and blue for *A. tumefaciens* KYC55 (Figure 1). H2 and H7 strains were only found to produce AHLs when assayed with *A. tumefaciens* KYC55 as the reporter strain (data not shown).

### 3.2. Identification of AHL-Producing Bacteria

**Figure 2:**
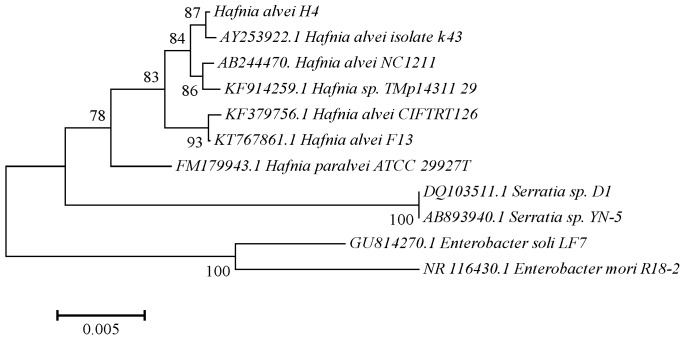
Phylogenetic analysis of AHL-producing bacteria isolated from spoiled ready-to-eat sea cucumber. Phylogenetic analysis was performed with MEGA4.1 software.

Comparison of the complete 16S rDNA gene sequences from the three bacteria with the sequences available in the NCBI database by nucleotide blast analysis indicated that H2, H4, and H7 belong to *Pesudomonas*, *Hafnia* and *Acinetobacter*, respectively. Since H4 appeared to have a stronger quorum sensing activity than the other two strains, it was subjected phylogenetic analysis. The phylogeny tree obtained by MEGA 3.1 software using the 16S rDNA sequences of 10 bacterial strains from NCBI database was presented as Figure 2. The result from the well-supported phylogeny with high resolution inner branches indicated that H4 was a *Hafnia alvei* strain, and therefore it was referred to as *H. alvei* H4. The AHLs extracted from *H. alvei* H4 culture were subjected to further analysis to identify the individual AHLs.

### 3.3. AHL Profile Analysis by TLC and HPLC-MS/MS

**Figure 3:**
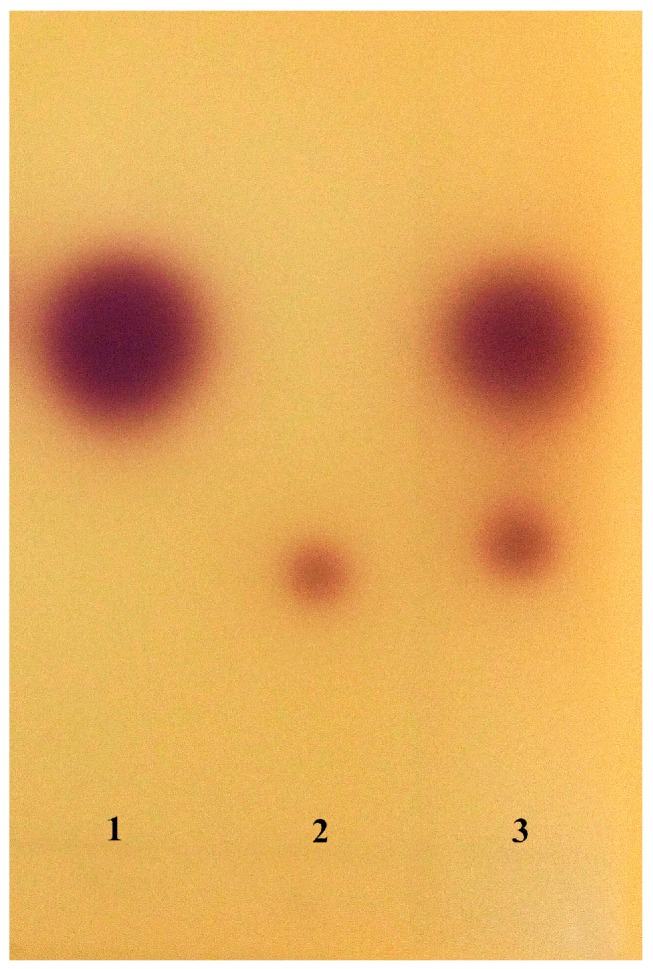
TLC bioassay of AHLs produced by *H. alvei* H4 with *C. violaceum* CV026 Lane 1: Standard AHLs of C4-HSL; Lane 2: Standard AHLs of C6-HSL; Lane 3: AHLs extracted from *H. alvei* H4 culture supernatant.

**Figure 4:**
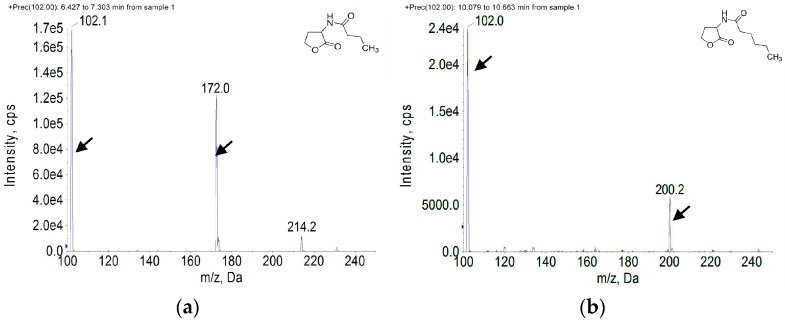

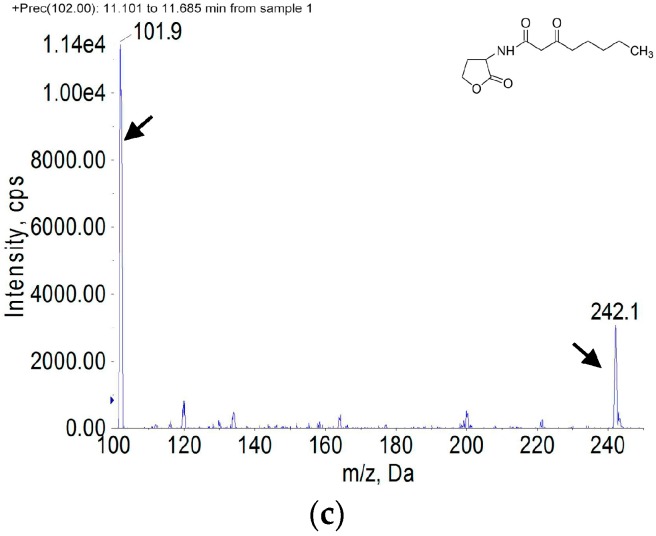
Mass spectrometry analysis of AHLs extracted from *H. alvei* H4 culture supernatant; (**a**) Spectrum of C4-HSL (*m*/*z* 172.0); (**b**) Spectrum of C6-HSL (*m*/*z* 200.2) (marked by arrow); (**c**) Spectrum of 3-oxo-C8-HSL (*m*/*z* 242.1) (marked by arrow).

TLC analysis of the AHLs extracts revealed two purple spots with the retention factors (Rf) corresponding to those of C4-HSL (Rf 0.62) and C6-HSL AHLs (Rf 0.38) (Figure 3). Further identification was conducted by HPLC/MS, comparison of the spectra (Figure 4 ) generated on the LC/MS platform compared to the standard AHLs revealed that *H. alvei* H4 produced three types of AHLs: C6-HSL (*m*/*z* 200), C4-HSL (*m*/*z* 172), and 3-oxo-C8-HSL (*m*/*z* 242).

### 3.4. AHLs Production by H. alvei H4 in Growth Phase

**Figure 5:**
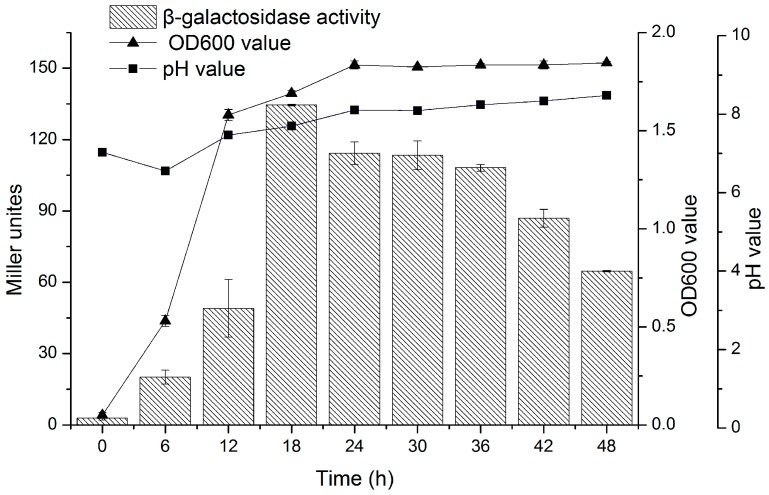
Production of AHLs by *H. alvei* H4 at different stages of growth in LB medium. AHLs in the culture supernatant of *H. alvei* H were quantitated by the β-galactosidase assay with *A. tumefaciens* KYC55 as the reporter strain.

*H. alvei* H4 produced AHLs throughout the 48 h of incubation in LB at 30 °C, with the level of AHLs reaching a maximum of 135 Miller unites after 18 h (Figure 5), and gradually decreased at late log and stationary phases to 110 Miller unites, followed by rapid decrease until 36 h, and down to 67 Miller unites at the end of 48 h. At the same time, the pH of the bacterial culture also increased gradually during incubation and reached 8.47 after 48 h. 

### 3.5. Effect of AHLs on Biofilm Formation of H. alvei H4

Growth of *H. alvei* H4 was not affected by the addition of AHLs as evidenced by a lack of change in OD_600_ values. Addition of C4-HSL to the culture significantly (*p* < 0.05) inhibited the biofilm formed by *H. alvei* H4, but only at low concentrations of C4-HSL (5 and 10 µM), since at high concentrations (20 and 40 µM) no significant difference was observed compared to the control. In contrast to the effect of C4-HSL, low concentrations of C6-HSL significantly (*p* < 0.05) promoted biofilm formation by *H. alvei* H4, although high concentration of C6-HSL slightly inhibited biofilm formation. 3-oxo-C8-HSL exhibited slight inhibition against the biofilm formed by *H. alvei* H4, with the greatest inhibition achieved by 5 µM 3-oxo-C8-HSL (*p* < 0.05).

## 4. Discussion

In this study, three bacteria were isolated from spoiled instant sea cucumber, and subsequently identified as members of the *Pesudomonas*, *Hafnia* and *Acinetobacter* genera by 16S rDNA analysis. All three strains are considered as food-spoilage bacteria [29]. *Pesudomonas* is widely present in chill-stored proteinaceous raw foods, vacuum-packed food and aquatic food products, but has also been isolated from spoiled pasteurized milk [6], whereas *Hafnia* is often found in vacuum-packed, refrigerated spherical fish paste and fresh meat [3,30]. *Acinetobacter* is found in brand beef and on food processing surfaces [31,32]. Quorun sensing system has been shown to play a role in bacteria-mediated food spoilage, especially those mediated by gram-negative bacteria, and the production of AHLs is thought to be an important contributing factor. Therefore, the bacterial strains isolated from spoiled instant sea cucumber were screened by *C. violaceum* CV026 and *A. tumefaciens* KYC55 for the ability to produce AHLs so that to determine their quorum sensing activity. H4 strain was found to secret AHLs as detected by both the two sensor strains, which presented a strong quorum sensing activity and subsequently identified as a *Hafina alvei* strain was chosen for further study. 

Three kinds of AHL molecules were detected in the culture supernatant of *H. alvei* H4, and these were identified as C4-HSL, C6-HSL, and 3-oxo-C8-HSL by the TLC and HPLC-MS methods (Figure 3 and Figure 4), and the charge-to-mass ratio (*m*/*z*) values of these detected compounds are consistent with the *m*/*z* values of C4-HSL, C6-HSL, and 3-oxo-C8-HSL reported by Ortori et al. [33]. A report has been published that the production of C6-HSL by *H. alvei* 071 isolated from cooled raw milk by Viana et al. [19], and 3-oxo-C8-HSL can be secreted by *H. alvei* FB1 as reported by Tan et al. [3], while the production of C4-HSL by *H. alvei* was demonstrated for the first time in this study*.* Spoilage of food caused by bacteria is mainly associated with the production of exoenzymes that cause the degradation of food ingredients. Production of proteolytic and lipolytic enzymes in *Serratia proteamaculans* B5a depends on the *lip*B gene, a process that is regulated by 3-oxo-C6-HSL as described by Christensen et al. [34]. Production of extracellular protease and biofilm formation were observed to be regulated by *ahyI*, which codes for AHLs synthase in *Aeromonas hydrophila*. A positive effect on these phenotypes was observed with a supplement of C4-HSL *to Aeromonas hydrophila ahyI* mutant*,* indicating the existence of a regulatory role for C4-HSL [35]. Both *H. alvei* and *Serratia* are members of the *Enterobacteriacea* family, and since *H. alvei* and *Aeromonas hydrophila* are opportunistic pathogens that are also closely related, a similar regulatory function of AHLs might exist in *H. alvei* as mentioned by Tan et al. [3]. However, this speculation needs to be verified by further study.

**Figure 6:**
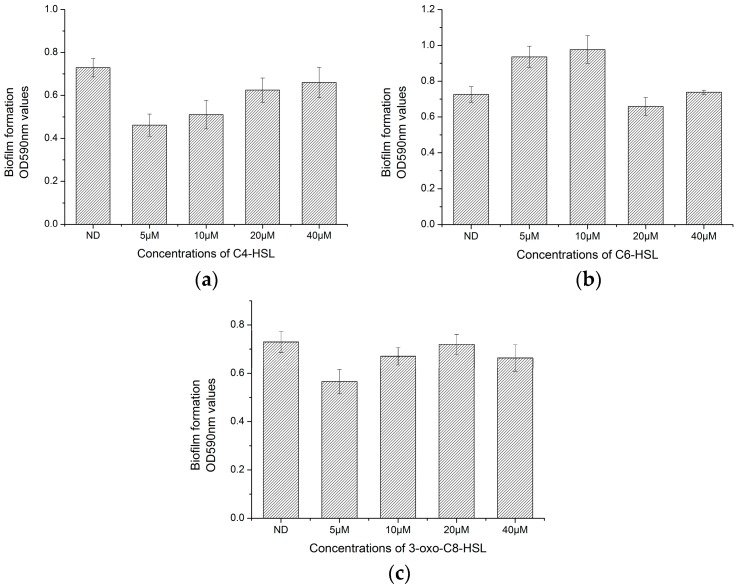
Effect of AHLs on the biofilm formation of *H. alvei* H4. (ND: No addition of AHLs). (**a**) Addition of 5–40 µM C4-HSL; (**b**) Addition of 5–40 µM C6-HSL; (**c**) Addition of 5–40 µM 3-oxo-C8-HSL.

In most Gram-negative bacteria, Quorum sensing is mediated by AHL molecules [4]. The trend in AHLs activity was consistent with the growth of *H. alvei* H4 within the first 18 h of incubation (Figure 6), since at late log phase, quorum sensing may play a more central role in bacterial physiology by slowing down its growth rate. This suggested that QS pathways may converge with starvation-sensing pathways, causing the cells to switch from the log phase to the stationary phase [36,37]. Thus the growth of *H. alvei* H4 might also be controlled by AHLs-mediated QS as explained by Nackerdien et al. [38] and Han et al. [36]. The reduction in AHLs activity that occurred when the pH of the culture became more alkaline was consistent with the fact that AHLs are unstable in alkaline condition [39].

To preliminarily verify the function of QS signaling molecules in *H. alvei* H4, the effect of each of the three AHLs produced by *H. alvei* H4 on its own biofilm formation appeared to vary, depending on the type of molecule. Except for C6-HSL, which promoted biofilm formation at low concentration, the other two all inhibited biofilm formation at low concentrations (Figure 6). Similar results were obtained by Nievas et al. [40], who showed that biofilm by *Peanut-Nodulating Bradyrhizobia* P8A strain can be inhibited by lower concentrations of (5 and 10 µM) 3-oxo-C10-HSL and 3-oxo-C14-HSL, consistent with the observed effects of C4-HSL and 3-oxo-C8-HSL on *H. alvei* H4 (Figure 6). In addition, the biofilm produced by *Peanut-Nodulating Bradyrhizobia* 62B strain can be increased by low concentrations of 3-oxo-C12-HSL (5 and 10 µM), also consistent with the result of C6-HSL on *H. alvei* H4 (Figure 6). A promoting effect was also observed on *Acinetobacter baumannii* ATCC19606 when C6-HSL was added [41]. Biofilm formation of *Serratia* A2 strain was inhibited by 10 µM C4-HSL and *Aeromonas* B1 strain was promoted by 10 µM C6-HSL reported by Zhang et al. [20], which were also in agreement to our finding. However, a contrary effect was obtained by Zhao et al. [16] when biofilm formation of *Shewanella baltica* was inhibited by 10 µM 3-oxo-C8-HSL, and biofilms of *Flavobacterium sp* and *Klebsiella sp1* was promoted by low concentrations of C4-HSL (10–100 nM) [42]. Therefore, considering the results achieved in this study and the description above, conclusions can be drawn that the effect of AHLs on biofilm formation varies to different bacteria, which were dose-dependent and type-dependent.

*LuxR*/*I* homologs in *H. alvei* are considered as *halR*/*I*. AHLs are synthesized by AHL synthase and regulated by *halI*. A *Hafnia alvei* 071 *halI* mutant that is unable to produce AHL is also deficient in biofilm formation [31]. This suggests an important role of AHLs in biofilm formation, which is linked to AHLs-mediated QS system*.* Although no direct evidence has been obtained to support a mechanistic relationship between QS and biofilm formation in *H. alvei* H4, the effect of AHLs on biofilm formed by *H. alvei* H4 was confirmed in our data.

## 5. Conclusions

Three spoilage bacteria were isolated from spoiled instant sea cucumber and identified as *Pesudomonas* H2, *Hafnia* H4, and *Acinetobacter* H7. Only *Hafnia* H4 exhibited strong AHL activity, and further identification showed that it was *H. alvei* H4 that produced three kinds of AHLs (C4-HSL, C6-HSL, and 3-oxo-C8-HSL). Biofilm formation *H. alvei* H4 could be significantly promoted or inhibited by the addition of the three kinds of AHLs with different concentrations. Further study will focus on constructing AHLs synthase mutant strain to research whether the production of extracellular protease and biofilm formation of *H. alvei* H4 were regulated by AHLs-mediated QS system.
